# Acute stroke CDS: automatic retrieval of thrombolysis contraindications from unstructured clinical letters

**DOI:** 10.3389/fdgth.2023.1186516

**Published:** 2023-06-14

**Authors:** Murray Cutforth, Hannah Watson, Cameron Brown, Chaoyang Wang, Stuart Thomson, Dickon Fell, Vismantas Dilys, Morag Scrimgeour, Patrick Schrempf, James Lesh, Keith Muir, Alexander Weir, Alison Q O’Neil

**Affiliations:** ^1^ Canon Medical Research Europe, Edinburgh, United Kingdom; ^2^ Institute of Neuroscience & Psychology, University of Glasgow, Glasgow, United Kingdom; ^3^ School of Engineering, University of Edinburgh, Edinburgh, United Kingdom

**Keywords:** clinical decision support (CDS), acute stroke, thrombolysis, machine learning (ML), named entity recognition (NER)

## Abstract

**Introduction:**

Thrombolysis treatment for acute ischaemic stroke can lead to better outcomes if administered early enough. However, contraindications exist which put the patient at greater risk of a bleed (e.g. recent major surgery, anticoagulant medication). Therefore, clinicians must check a patient's past medical history before proceeding with treatment. In this work we present a machine learning approach for accurate automatic detection of this information in unstructured text documents such as discharge letters or referral letters, to support the clinician in making a decision about whether to administer thrombolysis.

**Methods:**

We consulted local and national guidelines for thrombolysis eligibility, identifying 86 entities which are relevant to the thrombolysis decision. A total of 8,067 documents from 2,912 patients were manually annotated with these entities by medical students and clinicians. Using this data, we trained and validated several transformer-based named entity recognition (NER) models, focusing on transformer models which have been pre-trained on a biomedical corpus as these have shown most promise in the biomedical NER literature.

**Results:**

Our best model was a PubMedBERT-based approach, which obtained a lenient micro/macro F1 score of 0.829/0.723. Ensembling 5 variants of this model gave a significant boost to precision, obtaining micro/macro F1 of 0.846/0.734 which approaches the human annotator performance of 0.847/0.839. We further propose numeric definitions for the concepts of name regularity (similarity of all spans which refer to an entity) and context regularity (similarity of all context surrounding mentions of an entity), using these to analyse the types of errors made by the system and finding that the name regularity of an entity is a stronger predictor of model performance than raw training set frequency.

**Discussion:**

Overall, this work shows the potential of machine learning to provide clinical decision support (CDS) for the time-critical decision of thrombolysis administration in ischaemic stroke by quickly surfacing relevant information, leading to prompt treatment and hence to better patient outcomes.

## 1. Introduction

An acute stroke is a clinical emergency that requires prompt assessment and management. Around 85% of strokes are ischaemic (1), as opposed to haemorrhagic, requiring timely treatment by thrombolysis (intravenous clot busting medication) and/or thrombectomy (surgical mechanical clot retrieval). Not all ischaemic stroke patients are eligible for thrombolysis or thrombectomy; the decision is based upon historical and current patient factors, alongside imaging features. The remaining 15% of haemorrhagic strokes may require neurosurgical intervention but must not be treated with thrombolysis. We focus in this paper on thrombolysis; the faster the “door-to-needle time” with thrombolysis treatment, the better the chance of a good functional outcome for the patient (2). Current guidelines from the National Institute for Health and Care Excellence (NICE) state that treatment with a thrombolytic agent should be administered within 4.5 h post symptom-onset (3).

There are well-defined indications and, importantly, contraindications to thrombolysis that must be checked for all patients. Indications relate to the potential benefit of treatment, for instance, the pre- and post-stroke levels of independence. Contraindications to thrombolysis relate to the risk of bleeding, for instance, recent major surgery, anticoagulant medication, or a history of intracranial haemorrhage. Therefore, inappropriate treatment with thrombolysis can lead to catastrophic patient outcomes. The task of obtaining and reviewing all the relevant clinical information is complex and time-critical, leading to risks for both eligible and ineligible thrombolysis candidates, namely delayed treatment and missed contraindications respectively.

Information about indications and contraindications for thrombolysis comes from a variety of sources. Much of the information comes from the patient evaluation at the point of care, e.g. the patient history for the acute stroke event and the physical examination findings, as well as any immediate imaging results. However, a significant proportion of the required clinical information relates to the past medical history (e.g. recent major surgery, anticoagulant medication). The patient’s (electronic) health record can therefore be an important source of information, containing rich descriptions of past medical history in unstructured text documents such as discharge letters or referral letters. Automated surfacing of relevant information from the patient record could support the clinician in reviewing this information more quickly.

Named entity recognition (NER) is a well-studied information extraction task from the field of natural language processing (NLP). The task is to extract *spans*, i.e. subsections of a text, which refer to particular named entities. For example, in the general domain the entity set could be {person, location, organisation}. In our case, the list of relevant entities was compiled with reference to two sets of thrombolysis eligibility criteria: national guidelines from NICE (3) and a local checklist from Queen Elizabeth University Hospital (Table 1). This yielded a total of 86 entities, ranging from subarachnoid-haemorrhage to visual-disturbance. We classify the entities according to the following five categories: Diagnosis, Symptom, Social History, Medication, Treatment.

**Table 1:** The eligibility checklist for thrombolysis administration in use at Queen Elizabeth University Hospital, Glasgow, UK. This illustrates the scale of the criteria that must be satisfied and the information retrieval task required prior to administering thrombolysis.

The following must be Yes:	
Does the patient have symptoms of acute stroke?	Yes
Is there a measurable deficit on the NIH scale?	Yes
Was the patient previously independent?	Yes
Is there a clear time of onset within the last 4 ½ hours?	Yes
Has a CT scan since stroke onset excluded haemorrhage?	Yes
Has a senior member of the stroke team reviewed the CT scan?	Yes
The following must be No:	
Has the patient suffered head trauma or stroke within the last 3 months?	No
Has the patient undergone major surgery within the past 2 weeks?	No
Is there a past history of intracranial haemorrhage?	No
Is the history suggestive of SAH?	No
Is the systolic BP >185 mmHg (after treatment if necessary)?	No
Is the diastolic BP >110 mmHg (after treatment if necessary)?	No
Has there been any GI or urinary tract haemorrhage within the last 21 days?	No
Has there been an arterial puncture at a non compressible site within the last 7 days?	No
Was there a seizure at the time of symptom onset?	No
Is the patient on full dose anticoagulant treatment (e.g. warfarin with INR >1.5, therapeutic dose heparin/LMWH or oral thrombin inhibitor such as dabigatran, rivoroxaban)	No
If available, the answer should be No:	
Is the PT >15 sec (for those not on anticoagulants)?	No
Is the platelet count <100,000	No
Is the plasma glucose <2.7 or >22.2 mmol/l	No

**Figure 1:**
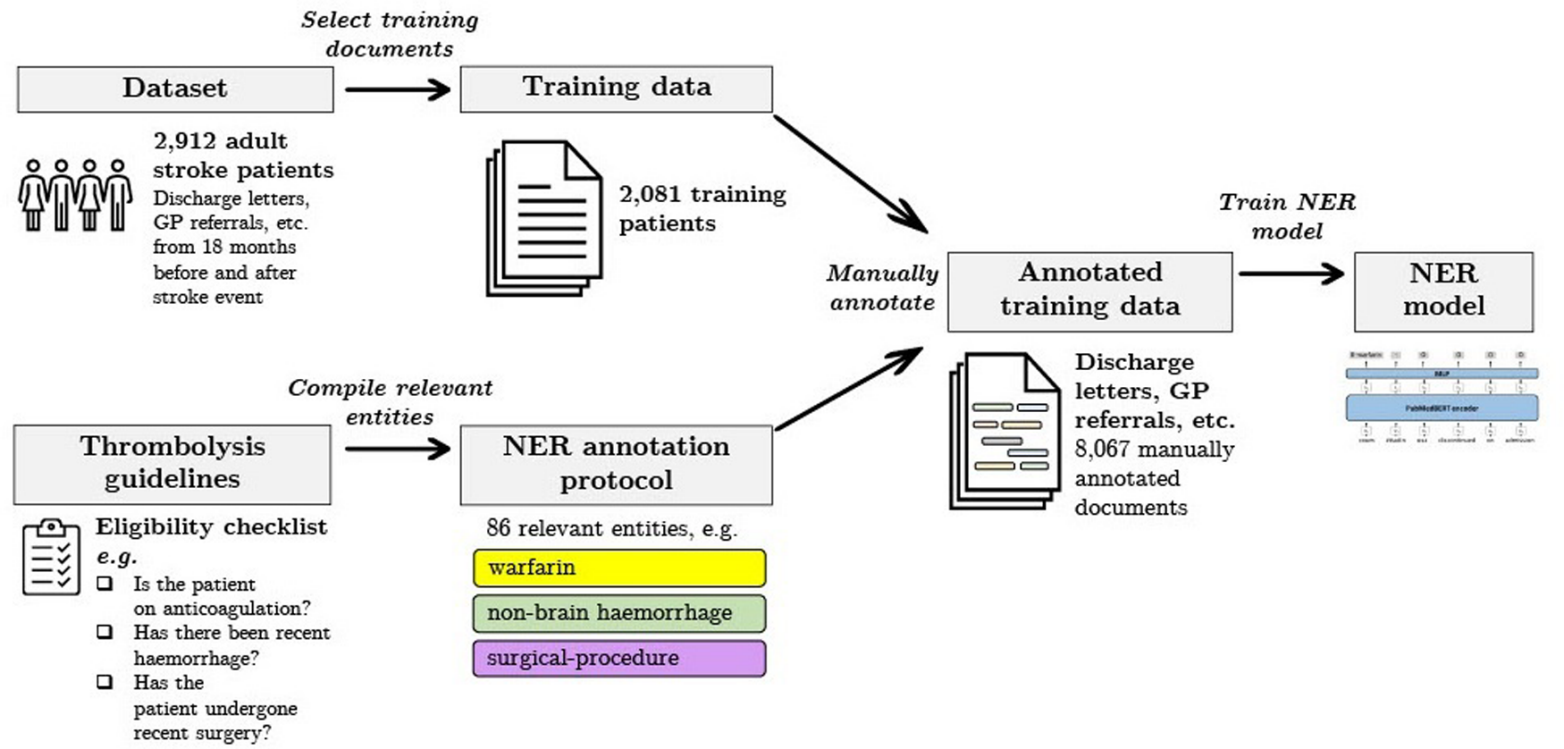
Summary of our data selection and annotation process. Our dataset contains discharge letters, clinic letters, GP referrals and endoscopy reports from 2,912 patients. The training set comprises 2,081 patients and the remainder are reserved for the held-out test set. Using both local and national guidelines, clinical knowledge was used to compile the relevant entities for thrombolysis decision. The NER model is trained to recognise 86 entities relevant to the thrombolysis eligibility checklist. Data was manually annotated by a team of clinicians and medical students.

**Figure 2:**
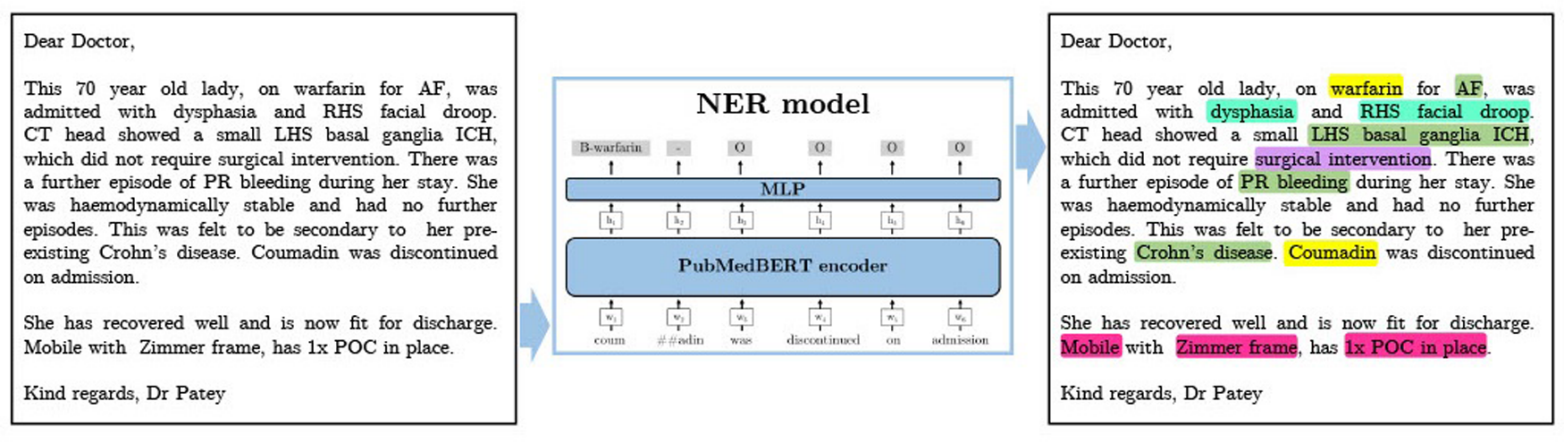
A synthesised discharge letter on which we demonstrate the application of our method. The output shows the entities that should be detected. Colour coding reflects category of entity (Green=Diagnosis, Turquoise=Symptom, Pink=Social History, Yellow=Medication, Purple=Treatment). AF, atrial fibrillation; ICH, intracranial haemorrhage; POC, package of care; PR, per rectum; RHS, right hand side; SAH, subarachnoid haemorrhage.

**Figure 3:**
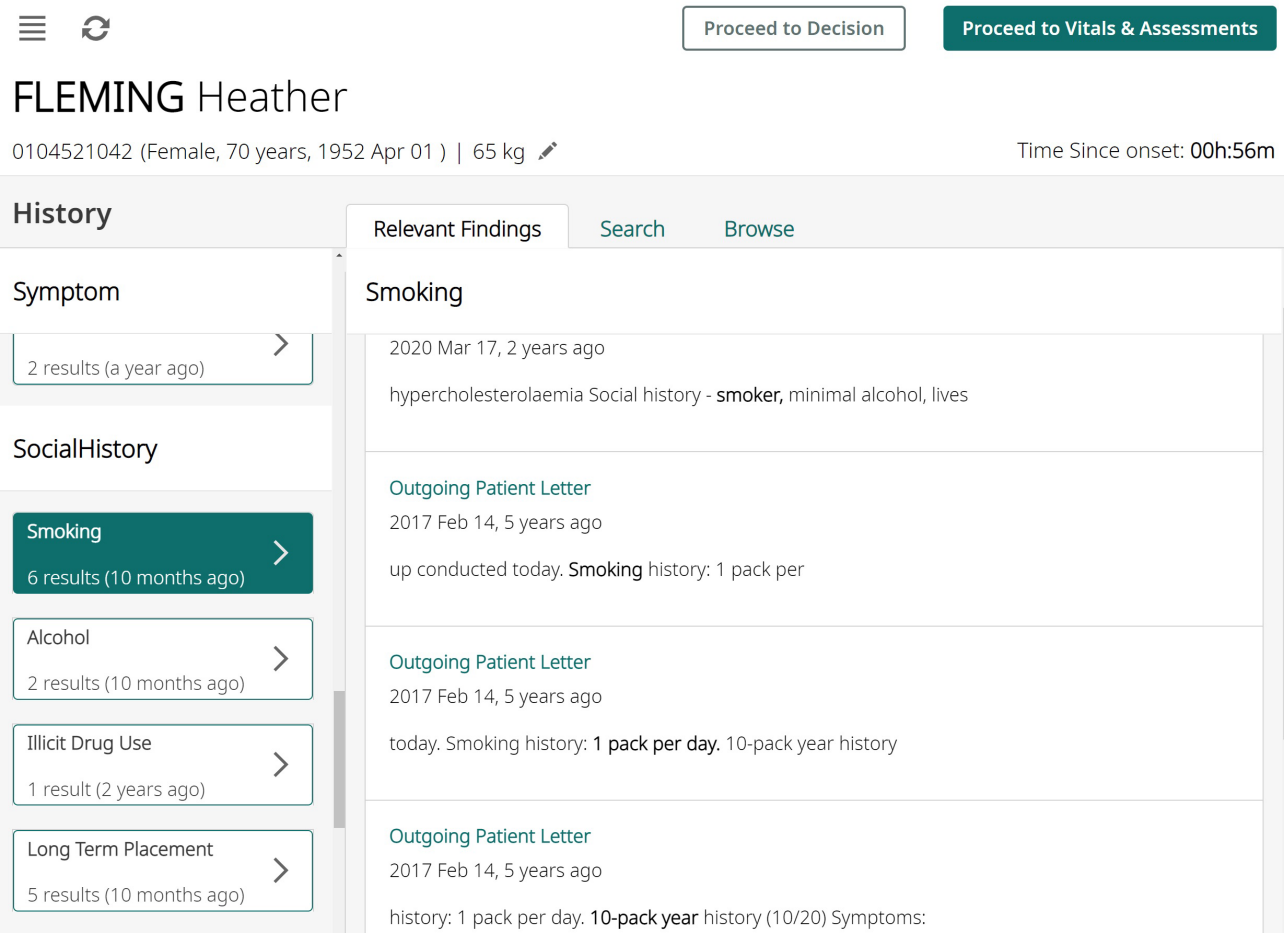
An illustration of how the algorithm results might be presented to clinicians using synthesised data. All entity occurrences are presented on the left pane, and then shown with context on the right pane, aiding rapid understanding of any contraindications.

In this work we measure and analyse the efficacy of transformer-based methods for NER in clinical text, training and validating on a large-scale dataset of unstructured clinical documents for the real-world problem of timely thrombolysis treatment in acute stroke patients. Data from almost 3,000 stroke patients was collected and annotated, as shown in the overview in Figure 1. We aim to evaluate if deep learning can provide accurate and robust performance for a clinical decision support (CDS) task. To illustrate the output of our work, Figure 2 shows the operation of our NER model on a synthesised discharge letter, and Figure 3 shows how the information from the NER model may be presented to the clinician to aid rapid understanding of contraindications.

### 1.1. Related work

We first review existing systems which aim to aid in thrombolysis CDS, and then subsequently review biomedical named entity recognition methods. In common with other NLP sub-fields, biomedical NER has advanced significantly in recent years due to the application of large pre-trained transformer architectures (4).

#### 1.1.1. Existing thrombolysis CDS systems for EHR data

Of the research groups working on CDS in the stroke domain, some identify and display contraindications to thrombolysis at the point of care (5, 6), whilst others focus on predicting outcomes in the case that thrombolysis is administered versus not administered (7–9). A recent review of machine learning methods for selecting patients who might benefit most from thrombolysis treatment is provided in (10). These methods are complimentary to our contraindication-finding approach. Most closely related to our approach is a small-scale feasibility study that designed a user interface to specifically highlight contraindications to thrombolysis by matching Unified Medical Language System (UMLS) (11) concepts between a thrombolysis eligibility checklist, and a stroke patient EHR (5). In contrast, our study is focused on the extraction of relevant concepts, and unlike (5) we use clinical domain expertise to curate a set of relevant entities and perform a detailed examination of the performance of various NER methods. To the best of our knowledge, none of the solutions described above are currently used in clinical practice.

#### 1.1.2. Biomedical named entity recognition

Biomedical NER is considered to be a slightly harder task than general domain NER due to the prevalence of abbreviations, synonymy, and morphological complexity as a result of the use of unusual characters such as Greek letters, digits, and punctuation (12). Clinical text poses an additional challenge, since clinicians frequently write in shorthand and may not always employ correct grammar. A number of public datasets exist which have allowed the development of different NER techniques, including JNLPBA (13), BC5CDR (14), NCBI (15), i2b2 (16), and MedMentions (17). Early methods included the application of dictionaries/gazetteers (18), or handcrafted features with probabilistic graphical models such as a conditional random field (19). The first generation of end-to-end deep neural architectures were based on character-level and/or word-level recurrent neural networks, often combined with a probabilistic graphical model to predict the final tag sequence, typified by the early work of (20).

Most recently, researchers in the biomedical NER field have focused on the application of large pre-trained transformer encoder models, based on Bidirectional Encoder Representations from transformers (BERT) (21). In this approach, a transformer model comprising encoder blocks only is pre-trained on a large corpus with two unsupervised NLP tasks: masked language modelling and next sentence prediction. A fully connected layer is then used to map the contextual word embeddings from the output of the BERT encoder to NER class logits, and the full model is fine-tuned using a supervised dataset. BERT-based NER approaches have been shown to outperform previous approaches (12).

#### 1.1.3. Extending BERT-based approaches for biomedical NER

The BERT-based NER approach has been extended in various ways. Some approaches aim to increase domain relevance. For instance, numerous studies have shown that biomedical NER tasks benefit from pre-training and vocabulary selection on a biomedical text corpus such as PubMed,^1^ yielding domain-specific models such as SciBERT (22), BioBERT (23), BioMed-RoBERTa (24), and PubMedBERT (25). Other studies have investigated integrating the Unified Medical Language System (UMLS) (11) biomedical knowledge graph with BERT architectures, such as (26–28), generally yielding a modest performance increase.

Other approaches target architectural improvements. For instance, BERT models have been combined with BiLSTMs in a two-stage proposal/refinement method in (29). Scaling up the model size and careful tuning of hyperparameters also gave modest improvements in (30). Finally, an encoder-decoder transformer architecture (text-to-text model) has shown strong performance on biomedical NLP tasks in (31).

Pre-training on biomedical data and using a biomedical vocabulary gives a consistent gain in performance. For example, on the NCBI-disease dataset, PubMedBERT (25) obtains an F1 of 87.8% versus the top scoring model with 90.4%, while general-domain BERT obtains 85.6%. On the BC5-chem dataset (14) PubMedBERT scores 93.3% versus the current top result of 95.0%, while general-domain BERT obtains 89.2%.

## 2. Materials and methods

### 2.1. Dataset

We use data obtained through a collaboration with the Industrial Centre for Artificial Intelligence Research in Digital Diagnostics (iCAIRD),^2^ for which we obtained ethical approval.^3^ The data was sourced from hospitals in the Greater Glasgow & Clyde (GG&C) area in Scotland and comprises all adult patients who were diagnosed with a stroke in the period 1 Jan 2013 to 31 Dec 2018. The data is pseudonymised and we accessed it onsite at the West of Scotland Safe Haven within NHS Greater Glasgow and Clyde via the Safe Haven Artificial Intelligence Platform (SHAIP) (32). Note that since we requested data for 18 months on either side of the stroke event, many of the text documents arose from other non-stroke clinical events.

This dataset contains approximately 50 K documents from 10 K patients. Documents were a mixture of General Practice referrals, Intermediate Discharge letters (IDLs), Final Discharge letters (FDLs), Outpatient clinic letters (OPCLs), Emergency Department letters, and Endoscopy reports. All documents comprise unstructured (free text) data, and the content was generally written by doctors, either general practitioners (GPs) or hospital doctors from various specialties encountered by patients during the 18-month period preceding and following the stroke event.

#### 2.1.1. Data split

For the purpose of the study, we annotated a subset of 8,067 documents from 2,912 patients, which we split at the outset into 2,081 training patients and a held-out test set of 831 patients. The training split was then randomly subdivided into five folds to be used for cross validation and ensembling.

#### 2.1.2. Data annotation

We designed an annotation protocol and then conducted an extensive annotation effort involving 8 medical students and 4 clinicians with 2–10 years of clinical experience each. Annotation was performed using the brat rapid annotation tool (33). All annotators attended a refresher teaching session on stroke and a training session on the protocol prior to beginning annotation. Additionally, throughout the process, annotators were able to ask questions regarding annotation work, with questions and answers made visible to all annotators.

Through the duration of the annotation period, we performed regular quality checks on each annotator by interleaving a common subset of documents through each annotator’s allocated folder of documents, and computing agreement with consensus gold standard annotations created by the two lead clinicians who developed the protocol and were responsible for ongoing updates (H.W. and C.B., with 5 years and 10 years of clinical experience respectively). Approximately 5% of the annotated documents were repeatedly annotated in this way. In Section 3.1, an analysis of the human annotator error relative to the gold standard is presented.

#### 2.1.3. Annotation protocol

The annotation protocol was collaboratively designed between Canon Medical Research Europe, the University of Glasgow and Deep Cognito (34) We identified which entities to annotate based on the local Queen Elizabeth University Hospital clinical guidelines and national clinical guidelines from NICE (3). Table 2 describes the full set of 86 entities which emerged from this process. Entities are grouped under 5 categories: **Diagnosis, Symptom, Social History, Medication, Treatment**.

**Table 2:** Exhaustive list of stroke-related entities that require annotation alongside their synonyms or equivalents as per the annotation protocol.

Entity type	Member entities
Diagnosis	Stroke, ischaemic stroke, intracranial haemorrhage, subarachnoid haemorrhage, extradural haemorrhage, subdural haemorrhage, brain haemorrhage unspecified, non-brain haemorrhage, transient ischaemic attack, head trauma, non-head trauma, trauma unspecified, dementia, delirium, hyperlipidaemia, diabetes mellitus, hypoglycaemia, ischaemic heart disease, myocardial infarction, hypertension, atrial fibrillation, congestive heart failure, carotid stenosis, small vessel disease, peripheral vascular disease, pericarditis, endocarditis, aneurysm, aortic dissection, arteriovenous malformation, intracranial neoplasm, gastrointestinal ulceration, acute pancreatitis, bleeding condition, clotting condition, pregnancy, demyelinating condition, peripheral nerve disorder, cerebral abscess, Todd’s paresis, epilepsy, functional neurological disorder, encephalitis, migraine
Symptom	Weakness, speech disturbance, visual loss, other visual disturbance, sensation loss, confusion, altered conscious level, fall, seizure, papilloedema, neck stiffness, fever, vomiting, dizziness, headache, vertigo, ataxia, other cerebellar dysfunction, fluctuating neurological symptoms
Social history	Smoking, alcohol, illicit drug use, long term placement, requires help, impaired mobility
Medication	Warfarin, clopidogrel, rivaroxaban, apixaban, dabigatran, edoxaban, aspirin, heparin, other anticoagulant, oestrogen containing drug, herbal remedy
Treatment	Surgical procedure, thrombolysis, mechanical thrombectomy, other invasive procedure, treatment escalation decision, capacity decision

The protocol aims to contain sufficient detail to ensure consistency between annotators. A list of synonyms is provided for each entity and these lists were updated over the course of the annotation process, e.g. for the entity congestive heart failure possible synonyms are “Left ventricular systolic dysfunction,” “LVSD,” “cardiac decompensation” and “LV dysfunction.” Instructions are also provided on how to determine the extent of the annotation text span, e.g. for the entity ischaemic stroke the span might be “Apparent R MCA infarct” i.e. omit the word “apparent” but contain the anatomical qualifiers. In addition, examples of correct and incorrect spans were provided to disambiguate difficult cases, e.g. for the entity Confusion a correct span would be “altered mental status” and an incorrect span would be “memory loss.”

#### 2.1.4. Annotation statistics

In total, 8,067 documents were annotated. A breakdown of the patient demographics and number of annotations by document type, data split, and entity category are presented in Tables 3–5. There is a 55%–45% train-test split at the level of the annotated documents. All document types were annotated approximately in proportion to their frequency in our dataset.

**Table 3:** Basic demographic statistics from our dataset.

	Train (%)	Test (%)	Total (%)
Male	870 (41.8)	303 (36.5)	1,173 (40.3)
Female	818 (39.3)	258 (31.0)	1,076 (37.0)
Unknown gender	393 (18.9)	270 (32.5)	663 (22.8)
Age (years) median (IQR)	78 (68-87)	77 (65-87)	78 (67-87)
**Ethnicity**			
White scottish	1,325 (63.7)	446 (53.7)	1,771 (60.8)
Unknown	636 (30.6)	342 (41.2)	978 (33.6)
Other white	83 (4.0)	30 (3.6)	113 (3.9)
Pakistani	17 (0.8)	4 (0.5)	21 (0.7)
Indian	9 (0.4)	3 (0.4)	12 (0.4)
Other	11 (0.5)	6 (0.7)	17 (0.6)
Unique patients	2,081	831	2,912

**Table 4:** Number of annotated documents (and corresponding unique patients), showing the prevalence of different document types in our dataset.

	Train	Test	Total
GP referral	2,465	1,938	4,403
IDL	733	1,118	1,850
FDL	1,082	392	1,474
OPCL	388	194	582
ED Letter	26	30	56
Endoscopy report	138	54	192
All document types	4,511	3,556	8,067
Unique patients	2,081	831	2,912

**Table 5:** Number of annotated entities. We labelled 86 different entities drawn from five categories, and instances are grouped in this table according to the entity categories.

	Train	Test	Total
Diagnosis	12,015	8,791	20,806
Symptom	6,309	5,230	11,539
Social history	5,430	3,994	9,424
Medication	7,248	6,143	13,391
Treatment	3,626	2,850	6,476
All entities	34,628	27,008	61,636

### 2.2. NER methodology

We now describe the NER algorithms which are evaluated in this work. Our main approach is based on a token-level transformer classifier.

#### 2.2.1. String search baseline

As a baseline, we have implemented a naive exact string search method. In this approach, the training data is used to compile a list of possible strings for every label (ambiguous cases are ignored). Predictions are then generated by searching for occurrences of each string, and then applying the corresponding label to that span.

#### 2.2.2. Transition model

A second, more sophisticated baseline is the transition-based model implemented in the spaCy library (35). This approach uses fixed word embeddings and a convolutional neural network, and is designed to be an effective and efficient general purpose NER method.

#### 2.2.3. Transformer models

The main approach applied to the NER problem in this work is a token-level classifier based on the BERT architecture (21). We used a number of different pre-trained weights and vocabularies from the literature: PubMedBERT (25) and BioMed-RoBERTa (24) are trained on biomedical papers from PubMed, while SciBERT (22) is trained on general scientific papers from Semantic Scholar). A separate label is assigned to each token in the model input by passing the contextual embedding of each input token to a multilayer perceptron (MLP) of 3 layers with 512 nodes. A probability distribution over the output classes is obtained by taking the softmax over the logits. In order to classify arbitrary length spans, an Inside-Outside-Beginning (IOB) tag scheme (36) is used. Under this scheme, the first token of an entity span should be assigned the B-*entity* tag and any subsequent tokens assigned I-*entity* tags, and any tokens not relating to entities should be assigned O tags. Therefore, given N entity classes, the model chooses from 2N+1 possible tags (in our case, 86 entities leads to 173 IOB classes). All trainable parameters in the model are optimised using the Adam optimiser (37) with a categorical cross-entropy loss function.

A number of model hyperparameters were explored, using a validation fold consisting of 20% of the training set. In Table 6 we present the optimal values which were found, as well as the search bounds. Where applicable, optimal parameters were found using the hyperopt Python package (39).

**Table 6:** Hyper-parameters used for individual BERT models.

Hyper-parameter	Value	Search space
Learning rate	$3\times 10^{-5}$	$\log_{10}(x)∼U(10^{-6},10^{-3})$
Train epochs	150	x∼U(50,200)
Batch size*	32	x∼U(1,64)
Weight decay*	0.01	$\log_{10}(x)∼U(10^{-3},10^{-1})$
LR schedule	Constant	{Constant, Linear, Cosine}
Loss function	Cross-entropy	{Cross entropy, Dice (38)}
MLP head depth	3	{1, 3, 5}
MLP head width	512	{512}

Rows marked with * were not found to significantly affect results, so default values were used.

#### 2.2.4. Ensemble of BERT models

Model ensembling is a well-known technique to obtain a modest performance improvement by taking the average prediction over a group of classifiers (40); ensembling causes random errors arising from individual classifiers to be smoothed out. We constructed an ensemble classifier using five variants of the best transformer model variant, trained on different subsets of the training set, according to a 5-fold cross validation split. The ensemble predictions were obtained by averaging the logits and taking the argmax over classes, allowing the confidence of individual models to be taken into account.

### 2.3. Evaluation metrics

In Section 3, we evaluate the performance of models using precision, recall, and F1. In the NER setting, these metrics can be computed in either a *strict* or *lenient* fashion. With strict matching, a predicted entity span must exactly match the ground truth span and label to count as a true positive, whereas with lenient matching the labels must match but a partial overlap between the spans is sufficient. For example, for the phrase “Patient on warfarin” (tokenised to [“patient,” “on,” “warfarin”]), the prediction [O, B-warfarin, I-warfarin] with ground truth [O, O, B-warfarin] would count as a lenient match but not a strict match.

### 2.4. Label properties

In order to better understand the performance of the NER methods on different labels, we examine three label properties, taking inspiration from the work in (41) which introduces the concepts of “name regularity” and “context regularity”. Here, we propose corresponding numeric definitions to measure each property for a given label in a given annotated dataset. The relationship between label properties and performance is presented in Section 3.2. The three properties examined for each label are:

1. *Training set frequency*. This is simply the number of instances of a particular entity label in the training set.
2. *Name regularity*. This is a measure of the similarity of all training set spans which are labelled as a particular entity, defined as:
  (1) $$\left(1-\frac{N_{\mathrm{unique}}}{N}\right)\frac{N}{N-1}$$
  where N is the total number of spans for the given label, and $N_{\mathrm{unique}}$ is the number of unique spans. The name regularity has a range of [0,1], taking a value of 1 when every span is identical, and a value of 0 when every span is different.
3. *Semantic context regularity*. This is a measure of the similarity of the context surrounding each entity mention. For each entity example, we take the 5 tokens on either side of the entity span, and compute the average of their word embeddings.^4^ For sample k, we call this mean embedding $x_{k}$. The semantic context similarity is then defined as:
  (2) $$\frac{2}{N(N-1)}\sum_{i=0}^{N}\sum_{\;j=i+1}^{N}S_{ij},\;\mathrm{where}\;S_{ij}=x_{i}\cdot x_{j}.$$
  In other words, this is a measurement of the similarity of the context around each entity mention, using the cosine similarity between the average of the word embeddings of the context. It has a range of [0,1], where a higher value indicates a more similar context.

## 3. Results

### 3.1. Quantitative evaluation

In Table 7, quantitative results on our held-out test set are presented. Uncertainty estimates were obtained by training 5 models using different random seeds and different training sets (using a five-fold cross validation split of the training set), and hence reflect the variance due to the model training procedure and due to the training set sample. Overall, these tables show that BERT-based models outperform our exact string search baseline, and that out of PubMedBERT (25), SciBERT (22), and BioMedRoBERTa (24), the PubMedBERT model performs best. A significant improvement in performance is observed using an ensemble of PubMedBERT models, particularly in the model precision.

**Table 7:** NER results on held-out test set using strict (upper table) and lenient (lower table) span matching, where strict matching requires an exact match of span boundaries and label, while lenient matching requires an exact match of label, and overlap in span boundaries. Best results indicated in bold. Estimated human annotation performance computed on a subset of 5% of documents shown for reference.

Method	Matching	Avg.	F1	Precision	Recall
String search			0.348	0.274	0.476
SpaCy transition-based (35)			0.710 ±0.003	**0.740 ±0.003**	0.682 ±0.008
SciBERT (22)			0.679 ±0.005	0.674 ±0.009	0.684 ±0.007
BioMed-RoBERTa (24)	Strict	Micro	0.690 ±0.003	0.681 ±0.007	0.699 ±0.002
PubMedBERT (25)			0.693 ±0.004	0.690 ±0.007	0.696 ±0.002
PubMedBERT ensemble			**0.722 ±0.001**	0.728 ±0.001	**0.715 ±0.002**
Human annotators			0.735	0.656	0.817
String search			0.416	0.416	0.489
SpaCy transition-based (35)			0.567 ±0.017	0.616 ±0.011	0.546 ±0.020
SciBERT (22)			0.609 ±0.003	0.617 ±0.015	0.620 ±0.007
BioMed-RoBERTa (24)	Strict	Macro	0.619 ±0.004	0.614 ±0.008	0.635 ±0.004
PubMedBERT (25)			0.622 ±0.008	0.629 ±0.007	0.628 ±0.014
PubMedBERT ensemble			**0.644 ±0.003**	**0.663 ±0.008**	**0.641 ±0.004**
Human annotators			0.727	0.678	0.817
Method	Matching	Avg.	F1	Precision	Recall
String search			0.547	0.432	0.745
SpaCy transition-based (35)			0.802 ±0.004	0.839 ±0.003	0.769 ±0.009
SciBERT (22)			0.815 ±0.003	0.810 ±0.008	0.819 ±0.009
BioMed-RoBERTa (24)	Lenient	Micro	0.826 ±0.002	0.817 ±0.006	0.837 ±0.002
PubMedBERT (25)			0.829 ±0.002	0.826 ±0.006	0.831 ±0.004
PubMedBERT ensemble			**0.846 ±0.001**	**0.854 ±0.001**	**0.837 ±0.002**
Human annotators			0.847	0.791	0.911
String search			0.580	0.577	0.690
SpaCy transition-based (35)			0.640 ±0.019	0.700 ±0.014	0.614 ±0.022
SciBERT (22)			0.713 ±0.003	0.725 ±0.012	0.722 ±0.009
BioMed-RoBERTa (24)	Lenient	Macro	0.721 ±0.005	0.719 ±0.011	0.738 ±0.004
PubMedBERT (25)			0.723 ±0.007	0.736 ±0.005	**0.728** ±0.014
PubMedBERT ensemble			**0.734 ±0.003**	**0.764 ±0.014**	**0.728 ±0.003**
Human annotators			0.839	0.803	0.910

For reference, we compare model prediction accuracy to the performance of our human annotators by comparing to the gold standard annotations on a common subset of documents which were annotated by all annotators. In total, there were 292 gold standard documents containing 2,870 spans. We can evaluate annotator performance on this set of documents using the quantitative metrics described in Section 2.3. Although the numbers are not directly comparable due to having been computed on different sets of documents, the ensemble model is approaching our estimate of human annotator micro F1. The ensemble model compares much less favourably in macro F1 to the human annotators, suggesting that the algorithm has difficulty learning how to label rare classes such as todds-paresis or peripheral-nerve-disorder.

The fact that the PubMedBERT-based models are approaching our estimate of the annotation error implies that quantitative evaluation using these annotations may not be reliable. In order to understand this issue, a further manual evaluation was undertaken by a clinician; results are presented in Section 3.3.

### 3.2. Error analysis

We further investigate the types of errors made by the PubMedBERT-based models (which performed best in Table 8), comparing to errors made by the baseline string search approach. Then, we examine the relationship between properties of individual labels and their performance. The large number of labels present in our NER problem means that the distribution of per-label scores carries information which we can analyse to understand what makes this NER problem difficult for the models under consideration.

**Table 8:** Multiple linear regression analysis for each model in which the independent variables are the three label properties, and the dependent variable is the lenient per–label F1. A scatter plot of each variable independent of the others is shown in Figure 5.

Method	Label property	β±SE	p	$r^{2}$
	$\log_{10}$(frequency)	0.077 ±0.043	0.076	
String search	name reg.	0.58 ±0.11	<0.001	0.39
	context reg.	-0.011 ±0.29	0.97	
	$\log_{10}$(frequency)	0.13 ±0.03	<0.001	
PubMedBERT	name reg.	0.58 ±0.08	<0.001	0.64
	context reg.	−0.016 0.19	0.93	
	$\log_{10}$(frequency)	0.16 ±0.03	¡0.001	
PubMedBERT ensemble	name reg.	0.54 ±0.08	<0.001	0.68
	context reg.	−0.06 ±0.19	0.72	

#### 3.2.1. Distribution of per-label F1 scores

**Figure 4:**
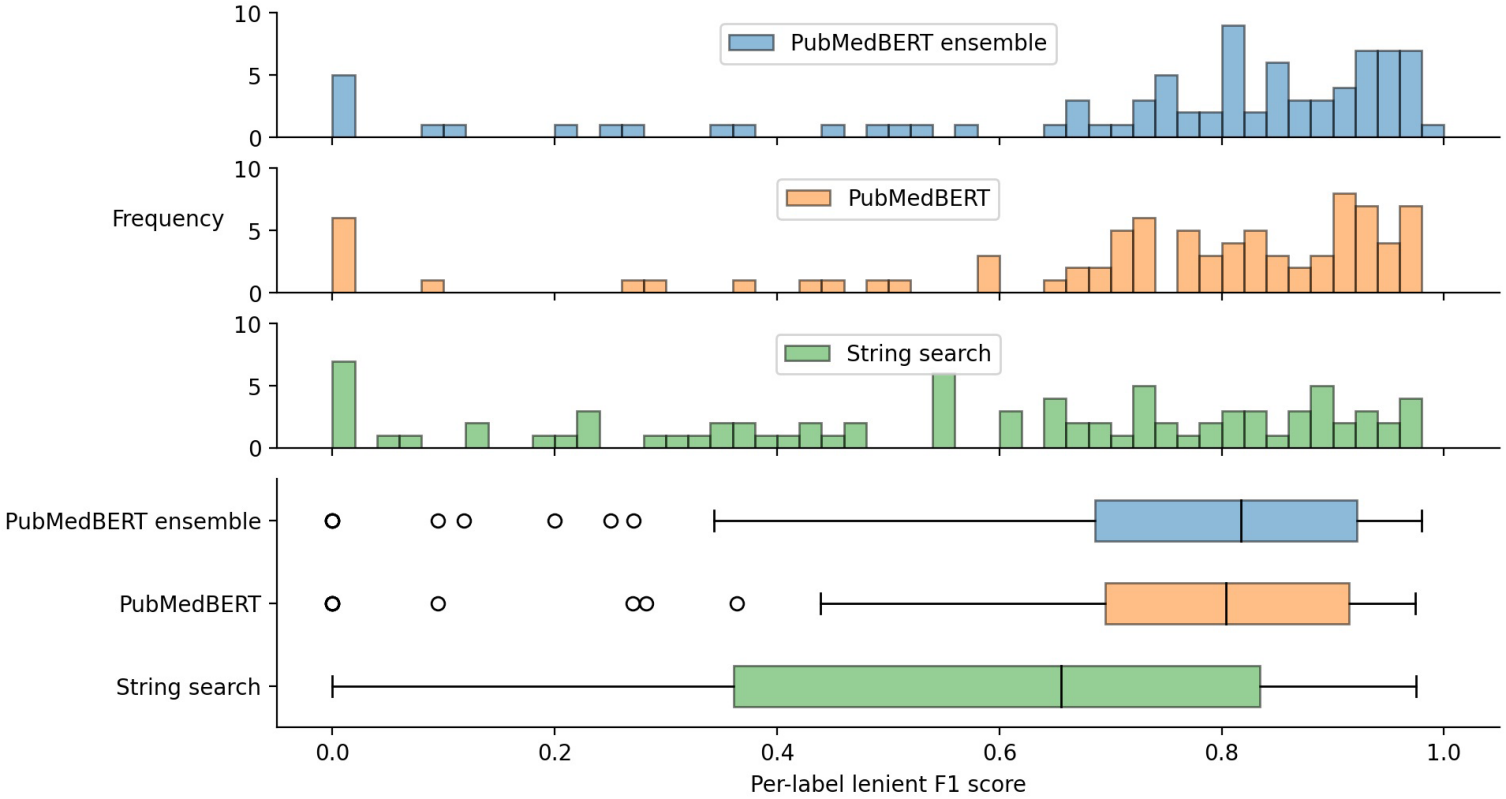
Box plot and histograms of per-label lenient F1 score.

The distribution of lenient F1 scores across each label is presented in Figure 4, showing that there is a relatively wide spread of performance between different labels. The best three labels for the ensemble method are aspirin (0.968), warfarin (0.965), and edoxaban (0.949), while the worst^5^ three labels are functional-neurological-disorder (0.095), cerebral-abscess (0.118), and neck-stiffness (0.2). Medications generally have a small set of possible surface forms, so these results suggest, as might be expected, that labels with fewer possible variants (i.e. higher *name regularity*) are easier to detect. Indeed, the best three labels have an average name regularity of 0.95, versus 0.17 for the bottom three.

#### 3.2.2. Effect of label properties on F1 score

**Figure 5:**
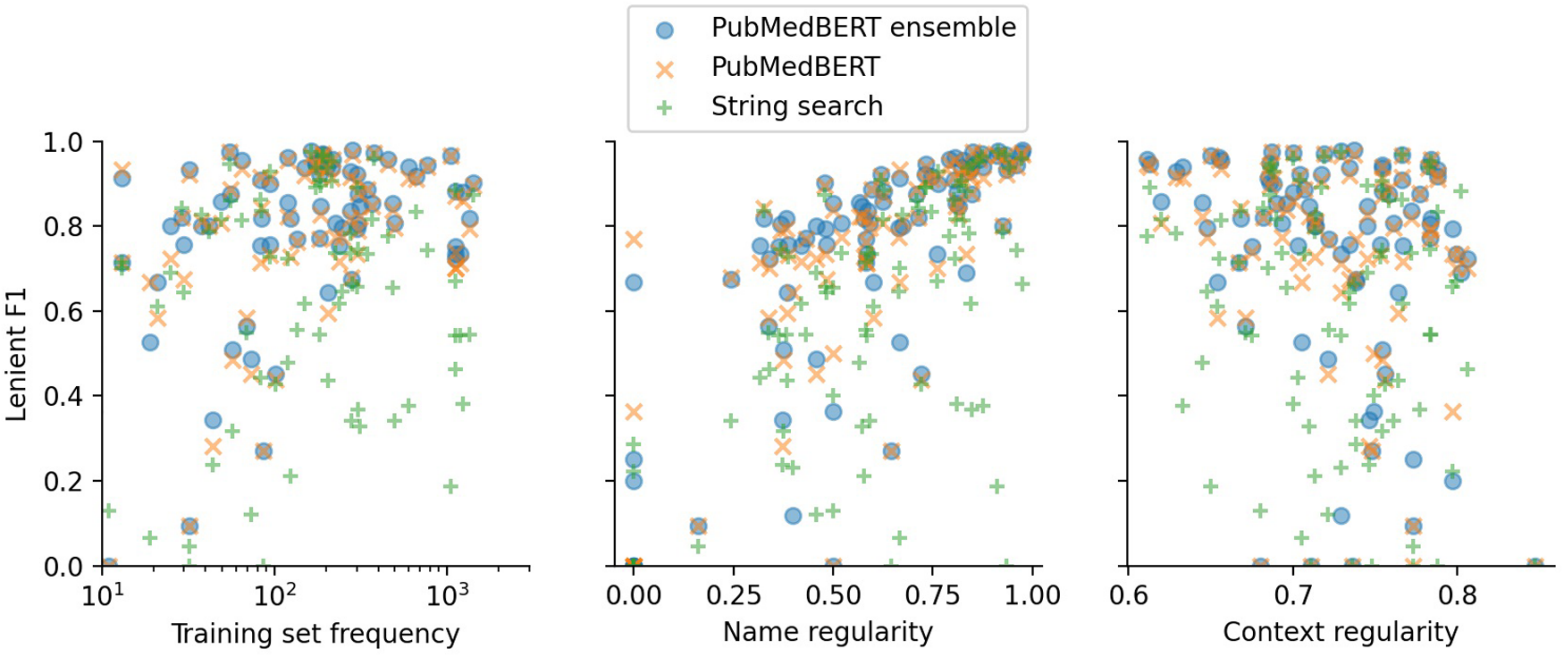
Lenient micro F1 versus different label properties. The y-value of each point shows the F1 score for a particular label using a particular model. Multiple linear regression analysis on this data is presented in Table 8.

In Figure 5 and Table 8 the effect of three label properties (described in Section 2.4) on performance for each model is investigated. Multiple linear regression was performed, treating the property as an independent variable and the per-label lenient F1 as the dependent variable. For all models, name regularity had the largest effect size. Suprisingly, the number of training set examples only had only a weak positive effect, and context regularity independent of performance. However, the relatively low $r^{2}$ values show that these three label properties are still insufficient to explain label performance.

### 3.3. Manual error analysis

Given that on certain metrics there is negligible difference between the estimate of human annotator error and the model error, the metrics computed against the ground truth may not reliably demonstrate clinically important differences. Therefore, in order to further investigate the differences in performance between the models, we conducted a manual clinical error analysis of the results.

#### 3.3.1. Error analysis protocol

A random subset of 310 documents from the held-out test set (this corresponds to approximately 10% of the test set) was selected for the manual error analysis. The corresponding human ground truth was then combined with the predictions from each model in turn, and the two sets of annotations were combined into a single view and displayed within our in-house text annotation software. A junior doctor (C.W.) with 3 years of clinical experience then reviewed all predictions, and either marked as correct, or if not then corrected as necessary. The evaluator was blinded to the source of the annotations (i.e. model prediction or human annotation) to reduce bias. This evaluation permits direct comparison between the precision of the model predictions and the human annotations, albeit on a much-reduced subset of the test set.

#### 3.3.2. Results of manual analysis

The manually-measured precisions are shown in Table 9. The precision of the ensemble model is notably much improved compared to the single PubMedBERT model with the number of pure false positive errors (type 1) decreased by two-thirds, and marginally better than the human annotators. The estimated precision of the human annotators is significantly higher than previously estimated using the gold standard documents in Section 3.1 (0.791 versus 0.944). This likely reflects the style of evaluation, where the evaluator is not making decisions independently of the annotator but rather rating visible annotations, and therefore in edge cases may tend to be generous. We also note that the evaluation document subsets are different so the metrics are not directly comparable.

**Table 9:** Manual evaluation of precision of selected models and human annotations on a subset of 310 documents from the test set.

Method	Matching	Avg.	Precision
PubMedBERT			0.803
PubMedBERT ensemble		Micro	**0.887**
Human annotators			0.871
PubMedBERT	Strict		0.764
PubMedBERT ensemble		Macro	**0.857**
Human annotators			0.836
PubMedBERT			0.888
PubMedBERT ensemble		Micro	**0.948**
Human annotators			0.944
PubMedBERT	Lenient		0.840
PubMedBERT ensemble		Macro	**0.914**
Human annotators			0.910

## 4. Discussion

Our results show that contemporary NER approaches are able to perform well on the task of locating relevant entities for the thrombolysis decision. Evaluation on the test set suggests that the ensemble of five PubMedBERT models achieves an almost identical F1 score to the human annotators (0.846 vs 0.847), which is exciting. However, further inspection shows the model has a better micro precision (0.854 vs. 0.791) but a worse micro recall (0.837 vs. 0.911) than the human annotators, which from a clinical point of view is not desirable, since false negatives are more serious than false positives when searching for contraindications. It is also notable that the micro and macro averages are similar for human annotators, whereas the machine-learning systems experience a drop of approximately 0.1 on macro averaged metrics, suggesting that rare labels are handled less effectively. However, the multiple linear regression analysis showed that in fact it was name regularity rather than training set frequency which played the most important role in determining performance i.e. rare *spans* are more problematic than rare labels. This point was reinforced by our observation that all transformer-based models experienced a significant drop in recall between the in-dict (i.e. spans which were also present in the training set) and out-dict portions of the test set (results not shown here). This suggests that name memorisation still plays a significant role for these models, and recognition of context or knowledge of synonymy learned during biomedical pre-training is not yet fully utilised. Name memorisation could be tackled through data augmentation techniques in which synonyms are gathered from knowledge sources such as UMLS (11). This type of data augmentation has already been implemented for NER with BERT models in (43), finding up to a 7% improvement in micro-F1, with the benefit falling off as the dataset size increased.

Compared to standard biomedical NER problems in the literature, this application features a large number (86) of fine-grained labels which allowed us to do inter-label analysis. It was shown that there is a large variation in performance between labels (inter-quartile range on per-label performance for ensemble model is 0.23). In the limiting case of many classes, this problem becomes very similar to entity linking—the task of linking entity mentions to knowledge graph nodes. A cross encoder refinement model from the two-stage models (44) which have been applied in entity linking may be useful to improve performance on some labels. The worst-performing labels may require special treatment, such as synthesized training data, in a future clinical application.

As previously stated, on some metrics the ensemble model obtained comparable performance to our estimate of the human annotator error. Manual analysis showed that the ensemble model predictions often surpass the original human annotations as measured by overall F1, but that the error types are different; human errors are more likely to be false negatives or protocol noncompliance, while model errors are more likely to be false positives. This suggests that review and improvement of the ground truth is required in order to make further improvements to the model, both to improve the standard of the training data and to improve our ability to identify and measure model improvements.

In this paper, we have considered only clinical free text data. However, structured data is also available both within the documents that we were working with and from other data types in the EHR. Information that might be expressed in structured format includes the patient’s current medications, diagnoses, recent procedures, or recent lab test results. Structured data is generally an easier and more standardised data format to parse (likely not requiring machine learning), and any clinical decision support system should consider this alongside the free-text data, integrating entity detections from both sources.

A limitation of this work is that the models were trained and evaluated on documents from both before and after the index stroke event. As the intended use for stroke CDS is on pre-stroke documents only, the performance in this context may change relative to the results in this work, however we believe that such a change is likely to be minor due to the similarity of the language between these cases, and the fact that 36% of the patients in our dataset experienced multiple strokes.

Finally, we remark that at present, our system does not directly answer the individual thrombolysis eligibility checklist items as this requires more information than just the existence of an entity. For instance, answering the question *“Has the patient undergone major surgery in the last two weeks?”* would involve first locating any occurrences of the surgery entity (using the NER model presented in this work), but also extracting any important modifiers which apply to each entity (e.g. negation and timeframe). The NER-only system presented here is a first step towards such a fully automated system, that leaves decision-making in the hands of the clinician who must judge for themselves the meaning of the surfaced information about relevant entities.

## 5. Conclusions

This work represents the first text-focused clinical decision support system for acute stroke treatment. Clinical guidelines were translated into a set of 86 entities relevant to the thrombolysis decision, and a large dataset of unstructured clinical letters of acute stroke patients was annotated for spans relating to these entities. Multiple transformer-based NER approaches were trained and evaluated. An ensemble of five PubMedBERT models obtained the best results (lenient micro F1 = 0.846/macro F1 = 0.734). This model was comparable to our estimate of human annotator performance (lenient micro F1 = 0.847/macro F1 = 0.839). One of the unusual aspects of our NER application was the large number of fine-grained labels, and a detailed error analysis showed that the name regularity was the strongest predictor of model performance on a given label. Finally, a further manual evaluation showed that the ensemble model outperformed a single PubMedBERT model by an even larger margin than suggested by the test set ground truth, due to annotation errors.

To the best of our knowledge, this work is the first text-focused decision support system for acute stroke treatment. Further, our system is the first step towards a clinical decision support system providing a recommendation for patients’ eligibility for thrombolysis in acute stroke care.

An important avenue for future work is to adopt a data-centric approach and develop review and correction techniques to improve the accuracy of human annotation ground truth. This will improve the standard of the training data and improve our ability to identify and measure model improvements, in order to enable further accuracy gains for this clinically critical task. Furthermore, in future work the entities extracted from unstructured data using the methods presented here should be integrated with entities extracted from any structured data which is already present, such as medication lists.

## Data Availability

The datasets presented in this article are not readily available because the data used in this study was obtained through the Industrial Centre for AI Research in Digital Diagnostics collaboration and comes from NHS Greater Glasgow and Clyde. It was accessed through the Canon Safe Haven Artificial Intelligence Platform tool. Due to patient confidentiality, the data used in this study is only accessible on application to the West of Scotland Safe Haven. Requests to access the datasets should be directed to https://icaird.com/.

## Footnotes

^1^ https://pubmed.ncbi.nlm.nih.gov/download/

^2^ https://icaird.com

^3^West of Scotland Safe Haven ethical approval number GSH19NE004.

^4^using the en_core_web_lg GloVe (42) word embeddings from the spaCy (35) package.

^5^Excluding 5 labels which the model failed to learn. These labels had F1 scores of 0, and on average appeared only 4.5 times each in the training set.
